# Supplementary material for: A Systematic Genotoxicity Assessment of a Suite of Metal Oxide Nanoparticles Reveals Their DNA Damaging and Clastogenic Potential
Source: Nanomaterials (Basel). 2024 Apr 24;14(9):743. doi: 10.3390/nano14090743 (PMC11085103; doi:10.3390/nano14090743)
Supplement: Supplementary file 1 [file nanomaterials-14-00743-s001.zip › Supplementary Information.pdf]

Supplementary Information

A systematic genotoxicity assessment of a suite of metal oxide nanoparticles reveals their DNA damaging and clastogenic potential

Silvia Aidee Solorio-Rodriguez, Dongmei Wu, Andrey Boyadzhiev, Callum Christ, Andrew Williams, Sabina Halappanavar*

Environmental Health Science and Research Bureau, Health Canada, Ottawa, Ontario, Canada.

*Corresponding author

Sabina Halappanavar, PhD

Environmental Health Science and Research Bureau, Health Canada, Ottawa, ON, Canada,

K1A 0K9

Email: sabina.halappanavar@hc-sc.gc.ca

Table S1. Sonication parameters for MONPs and MOMPs stock suspensions in dH_2O.

Stock suspension	Concentration	Volume	Sonication time	Amplitude	DSE (J/mL)
	(mg/mL)	(mL)	(min)	(%)	
Uncoated ZnO NPs (US3580)[a]	5	8	2.25	40	655
Uncoated ZnO NPs (5811HT)	5	50	14	40	655
APTES coated ZnO NPs (5812HT)	5	50	14	40	655
Stearic acid treated ZnO NPs (8412DL)	5	50	14	40	655
ZnO MPs (US1003M)[a]	5	8	2.25	40	655
Uncoated CuO NPs 544868[a]	1	8	1.9	10	109.5
Uncoated CuO NPs (US3070)	1	8	1.9	10	109.5
PVP coated CuO NPs (US3070)	1	8	1.9	10	109.5
Silane coated CuO NPs (US3070)	1	8	3.8	10	219
CuO MPs (US1140M)[a]	1	8	1.9	10	109.5
Uncoated MnO_2 NPs (4910DX)	5	8	1.5	40	440
MnO_2 MPs (4930DX)	5	8	1.5	40	440
Uncoated NiO NPs (US3355)	5	8	1.5	60	733
Uncoated NiO NPs (US3352)	5	8	1.5	60	733
PVP coated NiO NPs (US3352)	5	8	1.5	60	733
Stearic acid coated NiO NPs (US3352)	5	8	1.5	60	733
Silane coated NiO NPs (US3352)	5	8	3	60	1466
NiO MPs (US1014M)	5	8	1.5	60	733
Uncoated Al_2O_3 NPs (544833)	5	8	3	55	1270
Al_2O_3 MPs (1331DL)	5	8	3	55	1270
Uncoated CeO_2 NPs (US3136)	5	8	4	60	1953
Uncoated CeO_2 NPs (US3036)	5	8	4	60	1953
PVP coated CeO_2 NPs (US3037)	1	100	12	60	469
Stearic acid coated CeO_2 NPs (US3037)	1	100	12	60	469
CeO_2 MPs (2118CG)	5	8	4	60	1953
Uncoated TiO_2NPs[b]	5	50	15	55	1013
TiO_2 MPs (US1017M)[b]	5	50	15	55	1013
Uncoated Fe_2O_3 NPs (US3160)	1	100	10	55	338
Fe_2O_3 MPs (US1139M)	1	100	10	55	338

[a]Sonicator parameters published in [34]. [b]Previously published in [54]. Conditions were optimized from Avramescu et al. [38, 39]. The sonicator was operated for 8 seconds on with 2 seconds off. DSE: Delivered sonication energy. DSE = (P x T)/V. P=power (W), T=time (seconds), V=volume (mL).

Table S2. HD, PDI and ZP in dH_2O determined by DLS and ELS.

MONPs	H_2O		
	HD (nm)	PDI	ZP (mV)
Uncoated ZnO NPs (US3580)	271 ± 25.7	0.32 ± 0.05	21.1 ± 0.76
Uncoated ZnO NPs (5811HT)	255 ± 43.7	0.23 ± 0.04	21.52 ± 1.69
APTES coated ZnO NPs (5812HT)	408 ± 57.7	0.36 ± 0.05	14.4 ± 0.49
Stearic acid treated ZnO NPs (8412DL)	272 ± 43.5	0.31 ± 0.06	19.41 ± 0.49
Uncoated CuO NPs (544868)	647 ± 43.7	0.46 ± 0.06	−9.85 ± 0.54
Uncoated CuO NPs (US3070)	343 ± 21.4	0.39 ± 0.04	−9.85 ± 0.54
PVP coated CuO NPs (US3070)	309 ± 31.18	0.33 ± 0.05	11.9 ± 0.58
Silane coated CuO NPs (US3070)	552 ± 62.1	0.45 ± 0.06	36.9 ± 0.91
Uncoated MnO_2 NPs (4910DX)	134 ± 1.53	0.20 ± 0.03	−15.3 ± 1.71
Uncoated NiO NPs (US3355)	204 ± 10.1	0.31 ± 0.06	32.7 ± 3.70
Uncoated NiO NPs (US3352)	174 ± 2.38	0.21 ± 0.02	47.2 ± 0.67
PVP coated NiO NPs (US3352)	179 ± 1.44	0.16 ± 0.02	41.0 ± 0.69
Stearic acid coated NiO NPs (US3352)	260 ± 5.23	0.28 ± 0.03	38.0 ± 0.75
Silane coated NiO NPs (US3352)	199 ± 6.66	0.26 ± 0.04	48.1 ± 0.98
Uncoated Al_2O_3 NPs (544833)	350 ± 43.3	0.37 ± 0.06	29.5 ± 2.70
Uncoated CeO_2 NPs (US3136)	185 ± 7.85	0.29 ± 0.04	34.0 ± 1.05
Uncoated CeO_2 NPs (US3036)	189 ± 10.4	0.33 ± 0.05	−42.7 ± 3.63
PVP coated CeO_2 NPs (US3037)	410 ± 14.7	0.35 ± 0.05	33.0 ± 0.84
Stearic acid coated CeO_2 NPs (US3037)	399 ± 19.6	0.42 ± 0.06	−5.39 ± 1.16
Uncoated TiO_2 NPs	150.33 ± 2.08	0.14 ± 0.01	27.93 ± 0.96
Uncoated Fe_2O_3 NPs (US3160)	187 ± 15.5	0.33 ± 0.04	−38.6 ± 1.60

Table S3. % Solubility in DMEM cell culture media, and SSA of all MO particles.
NPs: nanoparticles. MP: microparticles. % Solubility data from Avramescu et al. [38, 41].

Particle (Catalogue Number)	% Solubility 10 μg/mL	% Solubility 100 μg/mL	SSA (m^2/g)
Uncoated ZnO NPs (US3580)	94.5	19.3	27.268
Uncoated ZnO NPs (5811HT)			21.715
APTES coated ZnO NPs (5812HT)			16.52
Stearic acid coated ZnO NPs (8412DL)			28.279
Uncoated ZnO MPs (US1003M)		11.8	5.85
Uncoated CuO NPs (544868)	12.6	51.6	10.343
Uncoated CuO NPs (US3070)			7.2
PVP coated CuO NPs (US3070)			8.779
Silane coated CuO NPs (US3070)			6.11
Uncoated CuO MPs (US1140M)		1.17	0.797
Uncoated MnO_2 NPs (SS4910DX)	4.79	3.87	42.164
Uncoated MnO_2 MPs (SS4930DX)		1.366	2.547
Uncoated NiO NPs (US3355)	0.94	1.81	36.602
Uncoated NiO NPs (US3352)			30.155
PVP coated NiO NPs (US3352)			36.675
Stearic acid coated NiO NPs (US3352)			14.629
Silane coated NiO NPs (US3352)			16.537
Uncoated NiO MPs (US1014M)		0.067	2.518
Uncoated Al_2O_3 NPs (544833)	1.11	0.73	145.292
Uncoated Al_2O_3 MPs (1331DL)		0.021	22.32
Uncoated CeO_2 NPs (US3136)			26.475
Uncoated CeO_2 NPs (US3036)	1.12	0.42	14.057
PVP coated CeO_2 NPs (US3037)			67.114
Stearic acid coated CeO_2 NPs (US3037)			32.854
Uncoated CeO_2 MPs (2118CG)			4.48
Uncoated TiO_2 NPs (NIST)	0.17	0.045	52.734
Uncoated TiO_2 MPs (US1017M)		0.0005	10.759
Uncoated Fe_2O_3 NPs (US3160)		0.0205	44.876
Uncoated Fe_2O_3 MPs (US1139M)			9.521
NPs included in BMC analysis			
**Particle (Catalogue Number)**			**SSA (m^2/g)**
Silica coated TiO_2 NPs (5422HT)			39.218
Silica and alumina coated TiO_2 NPs (5423HT)			28.607
Silica and stearic acid coated TiO_2 NPs (5424HT)			39.534
Silica and silicone oil coated TiO_2 NPs (5425HT)			17.541
Uncoated TiO_2 NPs (MKNA005)			150.100
Uncoated TiO_2 NPs (MKNA050)			62.937
Uncoated TiO_2 NPs (MKNR050P)			23.798

Table S4. Endpoint ranking based on μg metal/mL concentration normalized NOEC values. Where NOEC values were not present, LOEC values were used. Red: uncoated NPs. Green: coated NPs. Black: uncoated MPs. Blue: dissolved metal equivalent.

Metal	Comet 4 h (NOEC / LOEC Ranking)	Micronucleus 40 h (NOEC / LOEC Ranking)
Zn	ZnO US3580 ~ ZnO 5811HT ~ ZnO 5812HT ~ ZnO 8412DL > ZnO US1003M ~ $ZnCl_2$	ZnO US3580 ~ ZnO 8412DL > ZnO 5812HT > ZnO US1003M > $ZnCl_2$
Cu	CuO 544868 ~ CuO US3070 ~ CuO US3070P ~ CuO US3070Si > CuO US1140M ~ $CuCl_2$	CuO 544868 > CuO US3070Si ~ CuO US1140M > CuO US3070P > $CuCl_2$
Mn	$MnSO_4$ > MnO_2 4910DX > MnO_2 4930DX[(a)]	MnO_2 4910DX > $MnSO_4$ > MnO_2 4930DX[(a)]
Ni	NiO US3352St > NiO US3355 ~ NiO US3352 ~ NiO US1014M ~ NiO US3352Si > NiO US3352P > $NiCl_2$	NiO US3355 > NiO US1014M > NiO US3352St > $NiCl_2$ > NiO US3352P
Al	Al_2O_3 544833 ~ Al_2O_3 1331DL ~ $AlCl_3$	$AlCl_3$ > Al_2O_3 544833 > Al_2O_3 1331DL
Ce	CeO_2 US3136 ~ CeO_2 US3036 ~ CeO_2 US3037P ~ CeO_2 2118CG ~ $CeCl_3$ > CeO_2 US3037St	$CeCl_3$ > CeO_2 US3037St > CeO_2 US3036 ~ CeO_2 US3037P ~ CeO_2 2118CG
Ti	TiO_2 5423HT ~ TiO_2 MKNR050P > TiO_2 NIST ~ TiO_2 5422HT ~ TiO_2 5424HT ~ TiO_2 5425HT ~ TiO_2 MKNA005 ~ TiO_2 MKNA050 ~ TiO_2 US1017M	TiO_2 NIST ~ TiO_2 US1017M[(b)]
Fe	Fe_2O_3 US3160 ~ Fe_2O_3 US1139M	Fe_2O_3 US3160 > Fe_2O_3 US1139M[(b)]

[(a)]: Ranking is biased due to low concentration range for $MnSO_4$.
[(b)]: Interference seen.
TiO_2 NIST NPs is also referred as “uncoated TiO_2 NPs” in the main text.

Table S5. Endpoint ranking based on cm^2 particle / cm^2 well-plate concentration normalized NOEC values. Where NOEC values were not present, LOEC values were used. Red: uncoated NPs. Green: coated NPs. Black: uncoated MPs.

Metal	Comet 4 h (NOEC / LOEC Ranking)	Micronucleus 40 h (NOEC / LOEC Ranking)
Zn	ZnO US1003M > ZnO 5812HT > ZnO 5811HT > ZnO US3580 > ZnO 8412DL	ZnO US1003M > ZnO US3580 > ZnO 8412DL ~ > ZnO 5812HT
Cu	CuO US3070Si > CuO US3070 > CuO US1140M > CuO US3070P > CuO 544868	CuO US1140M > CuO 544868 > CuO US3070Si > CuO US3070P
Mn	MnO_2 4930DX > MnO_2 4910DX	MnO_2 4910DX ~ MnO_2 4930DX
Ni	NiO US1014M > NiO US3352St > NiO US3352Si > NiO US3352 > NiO US3352 > NiO US3352P	NiO US1014M > NiO US3355 > NiO US3352St > NiO US3352P
Al	Al_2O_3 1331DL > Al_2O_3 544833	Al_2O_3 1331DL > Al_2O_3 544833
Ce	CeO_2 2118CG > CeO_2 US3036 > CeO_2 US3136 > CeO_2 US3037St > CeO_2 US3037P	CeO_2 2118CG > CeO_2 US3036 > CeO_2 US3036St > CeO_2 US3036P
Ti	TiO_2 US1017M > TiO_2 MKNR050P > TiO_2 5423HT > TiO_2 5425HT > TiO_2 5422HT > TiO_2 5424HT > TiO_2 NIST > TiO_2 MKNA050 > TiO_2 MKNA005	TiO_2 US1017M > TiO_2 NIST[(a)]
Fe	Fe_2O_3 US1139M > Fe_2O_3 US3160	Fe_2O_3 US1139M > Fe_2O_3 US3160[(a)]

[(a)]: Interference seen.

TiO_2 NIST NPs is also referred as "uncoated TiO_2 NPs" in the main text.

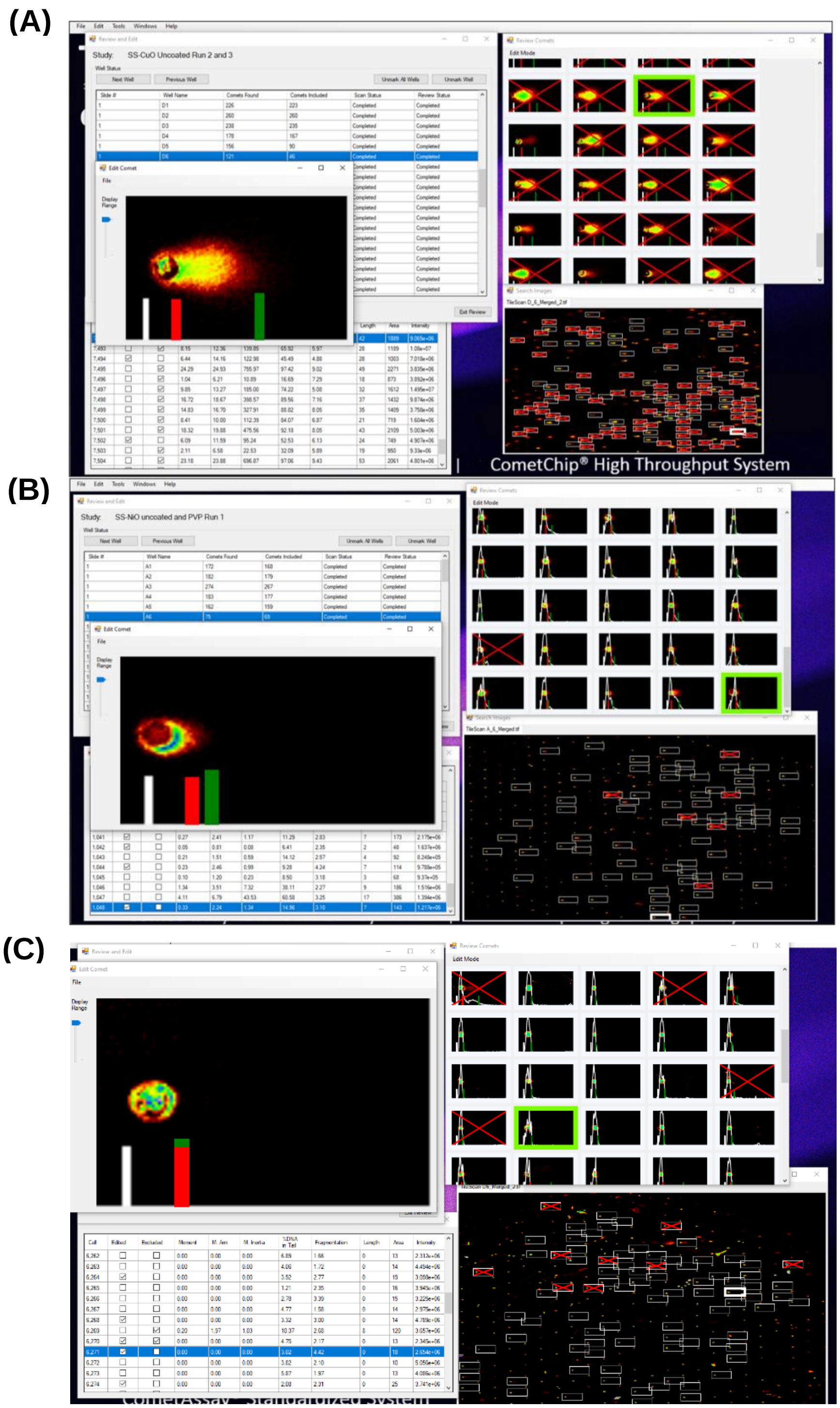

Figure S1. Representative images of the interference of some MONPs with the SYBR® Gold staining and the analysis of the comet assay at high concentrations. (A) Uncoated CuO NPs (US3070). (B) Uncoated NiO NPs (US3352). (C) Uncoated Fe_2O_3 NPs (US3160). Analysis was conducted using the Trevigen Comet Software.

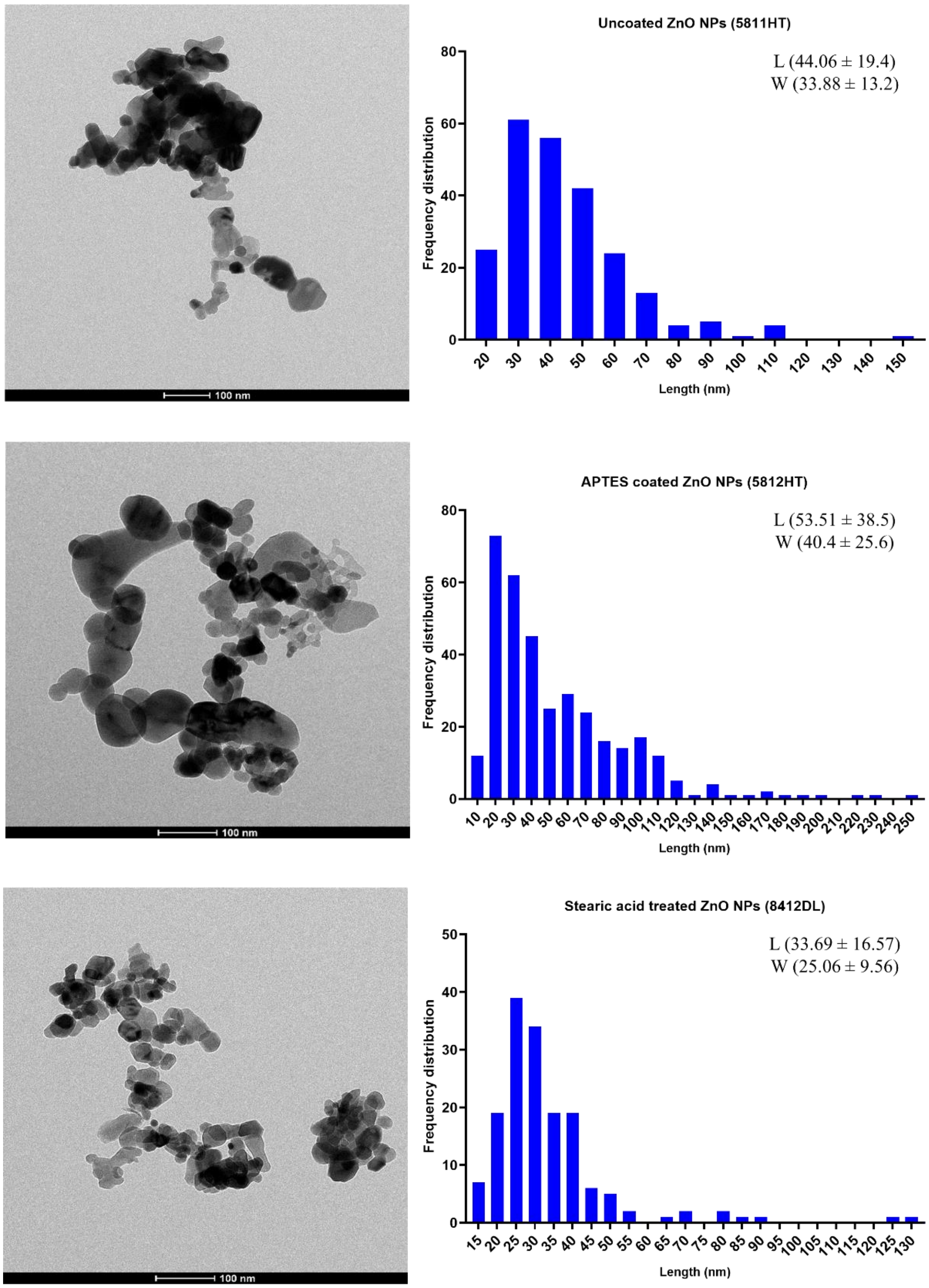

Figure S2. Representative TEM images and frequency size distribution of ZnO NPs variants. L: Length, W: Width. Scale bar: 100 nm.

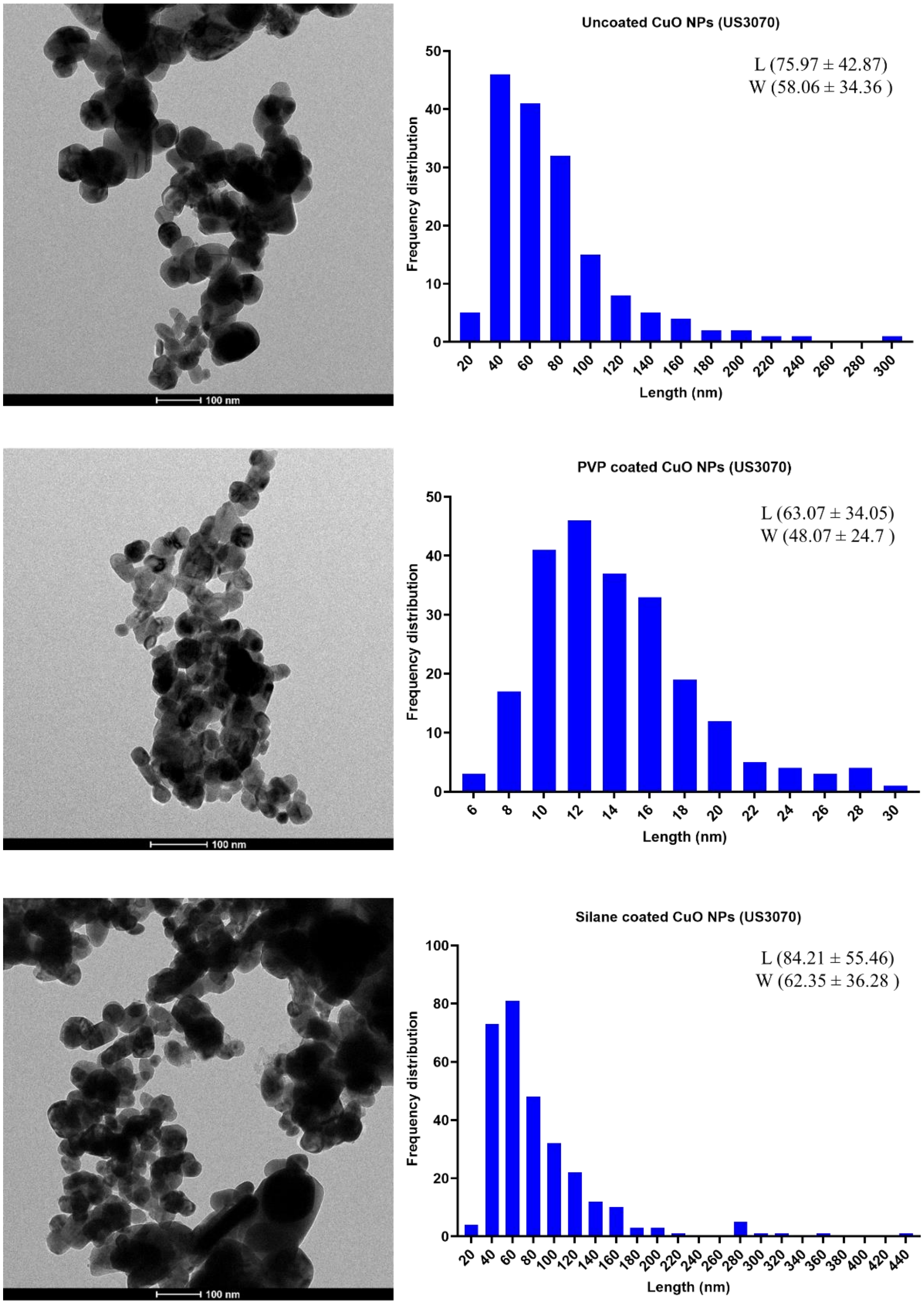

Figure S3. Representative TEM images and frequency size distribution of CuO NPs variants. L: Length, W: Width. Scale bar: 100 nm.

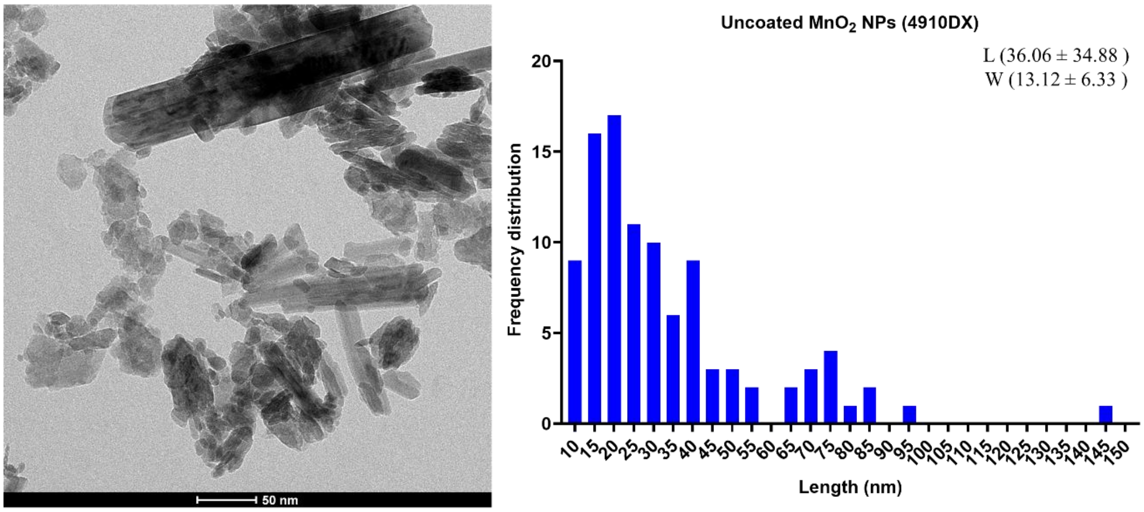

Figure S4. Representative TEM image and frequency size distribution of MnO_2 NPs. L: Length, W: Width. Scale bar: 50 nm.

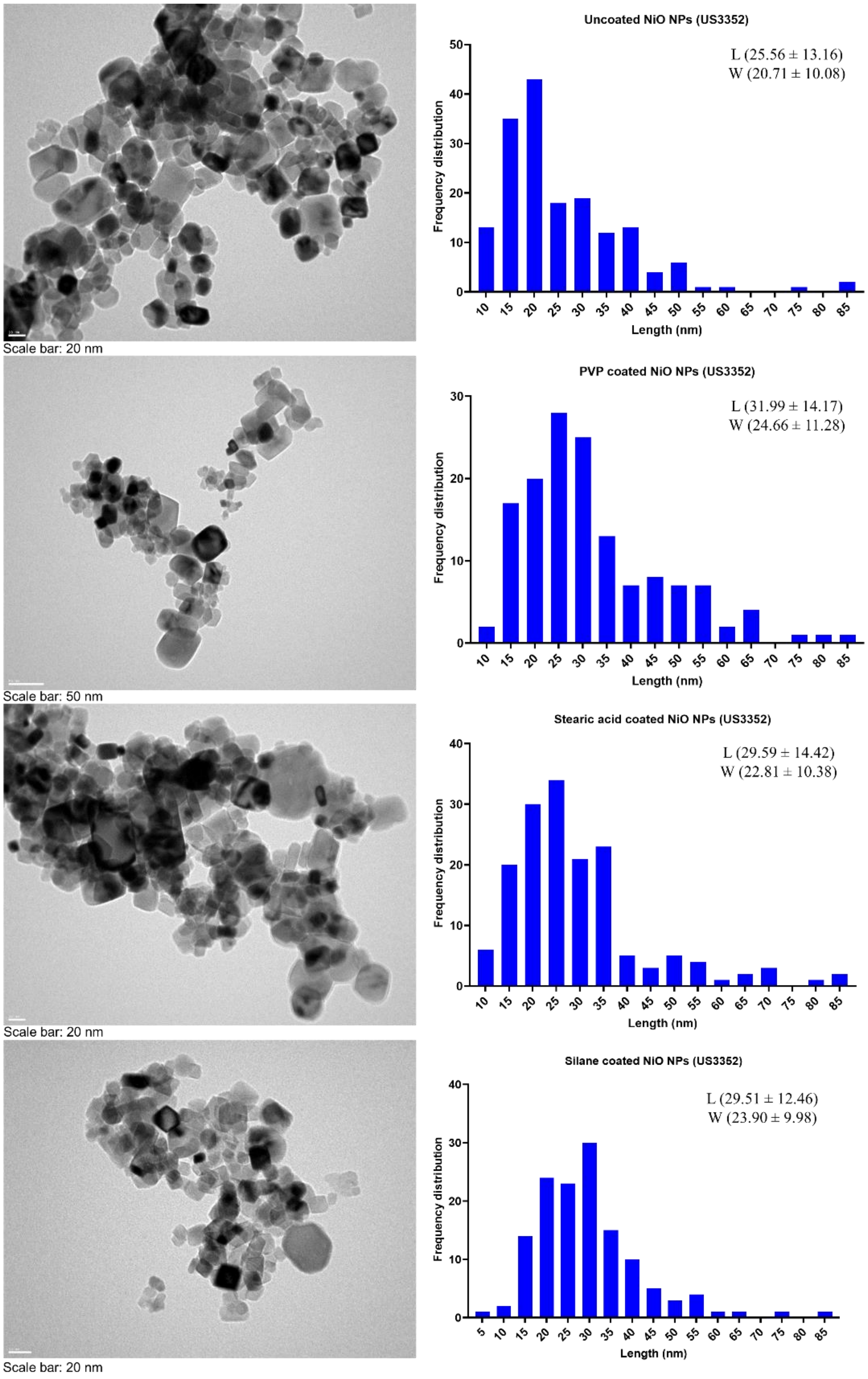

Figure S5. Representative TEM images and frequency size distribution of NiO NPs variants. L: Length, W: Width.

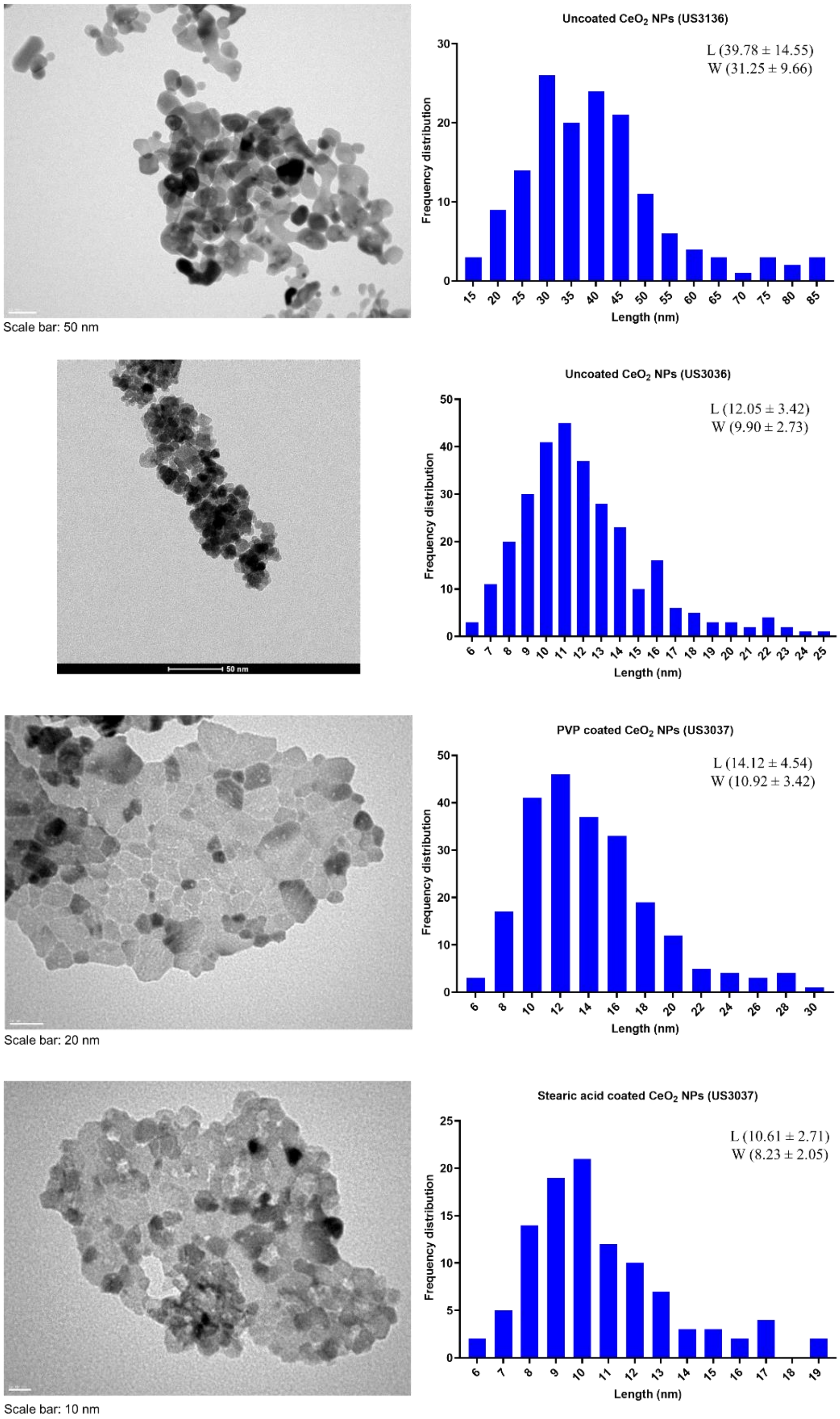

Figure S6. Representative TEM images and frequency size distribution of CeO_2 NPs variants. L: Length, W: Width.

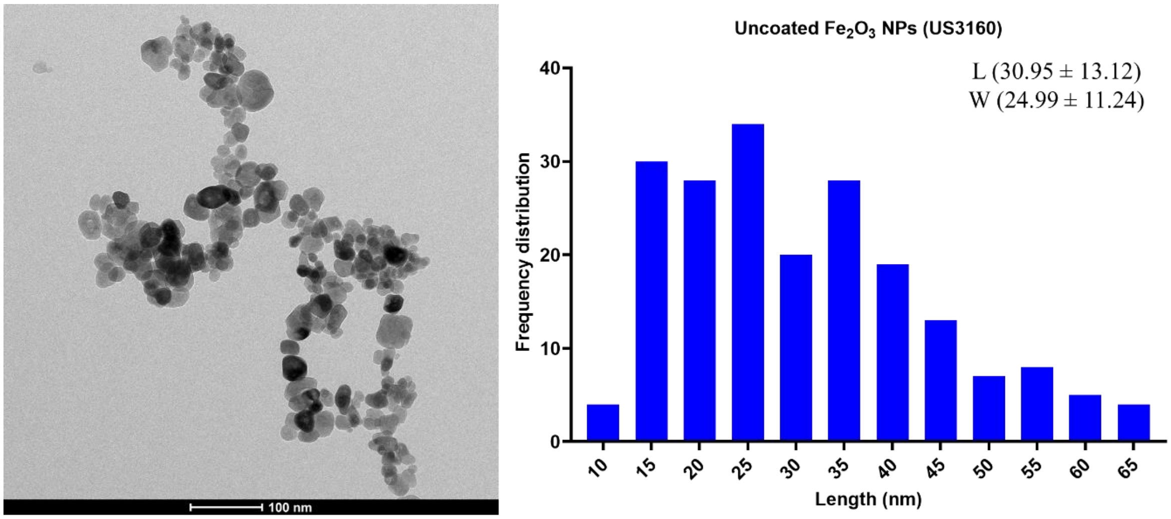

Figure S7. Representative TEM images and frequency size distribution of Fe_2O_3 NPs. L: Length, W: Width. Scale bar: 100 nm.

MnO_2 4930DX MOMPs

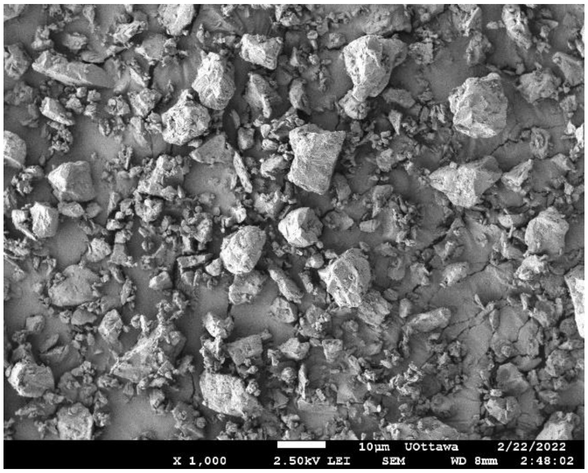

CeO_2 2118CG MOMPs

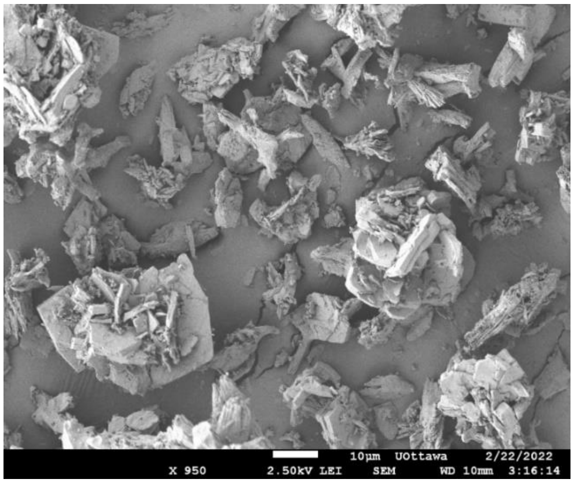

Fe_2O_3 US1139M MOMPs

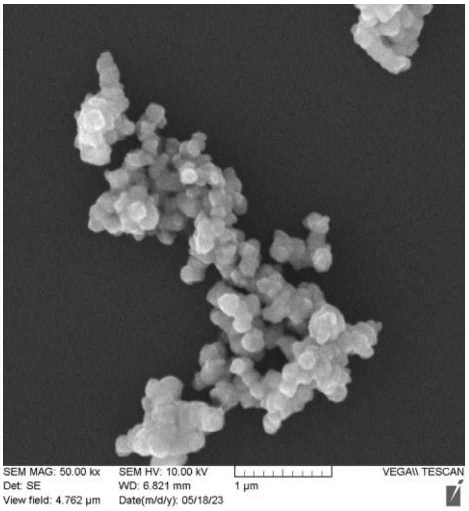

Figure S8. Representative SEM images of MnO_2 MPs, CeO_2 MPs and Fe_2O_3 MPs.

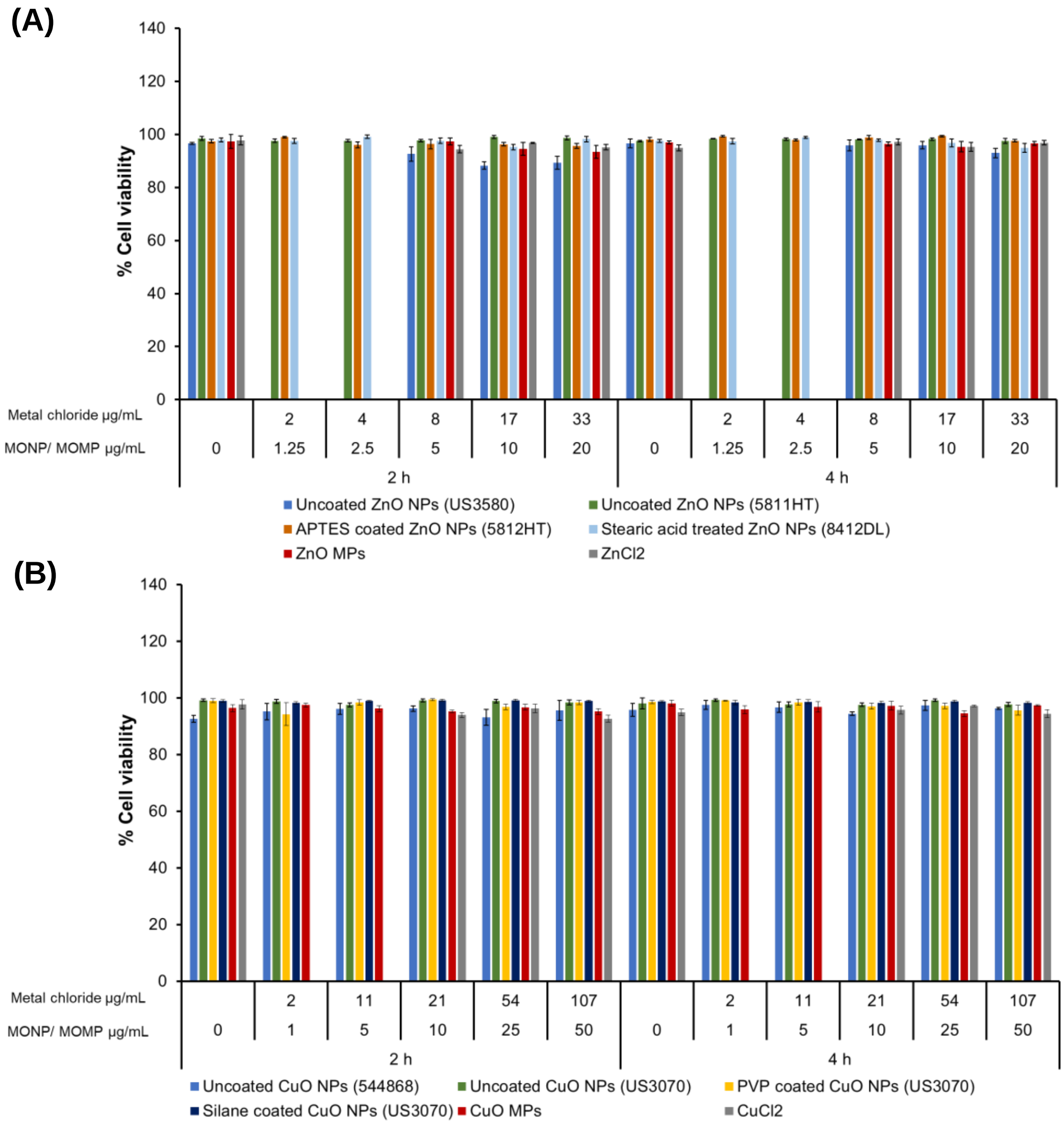

Figure S9. Percentage of cell viability after exposure to (A) ZnO variants and $ZnCl_2$, (B) CuO variants and $CuCl_2$. Trypan Blue exclusion method was conducted after 2 and 4 h of exposure. Data is presented as mean and standard error (n=3-4 independent experiments). Statistically significant differences between the exposed samples and the matched negative control were determined through Kruskal-Wallis test. Uncoated ZnO NPs (US3580), ZnO MPs, $ZnCl_2$, uncoated CuO NPs (544868), CuO MPs, and $CuCl_2$ were previously published in Boyadzhiev et al. [34].

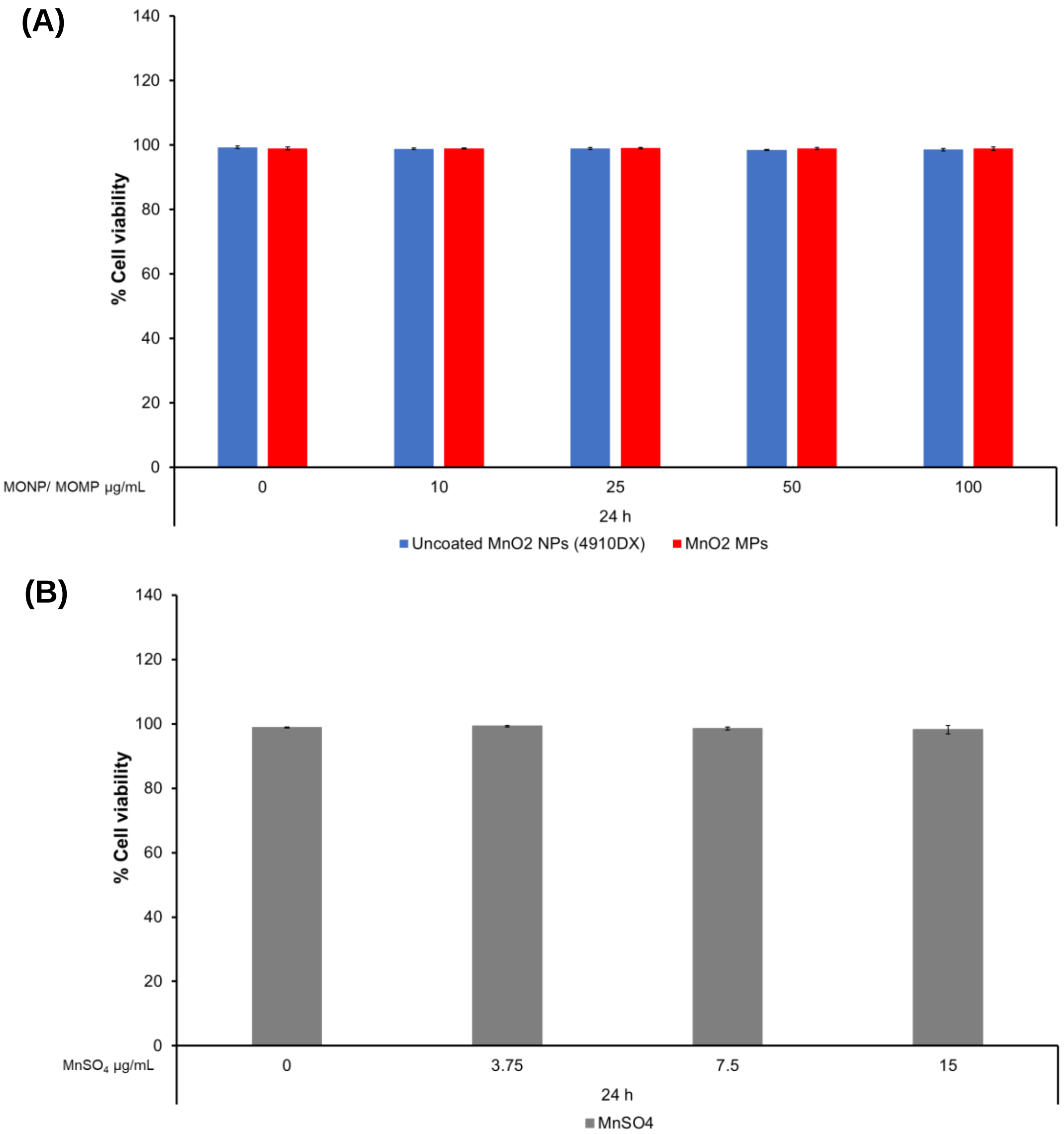

Figure S10. Percentage of cell viability after exposure to (A) MnO_2 variants and (B) $MnSO_4$ for 24 h. Trypan Blue exclusion method. Data is presented as mean and standard error (n=3-4 independent experiments). Statistically significant differences between the exposed samples and the matched negative control were determined through Kruskal-Wallis test.

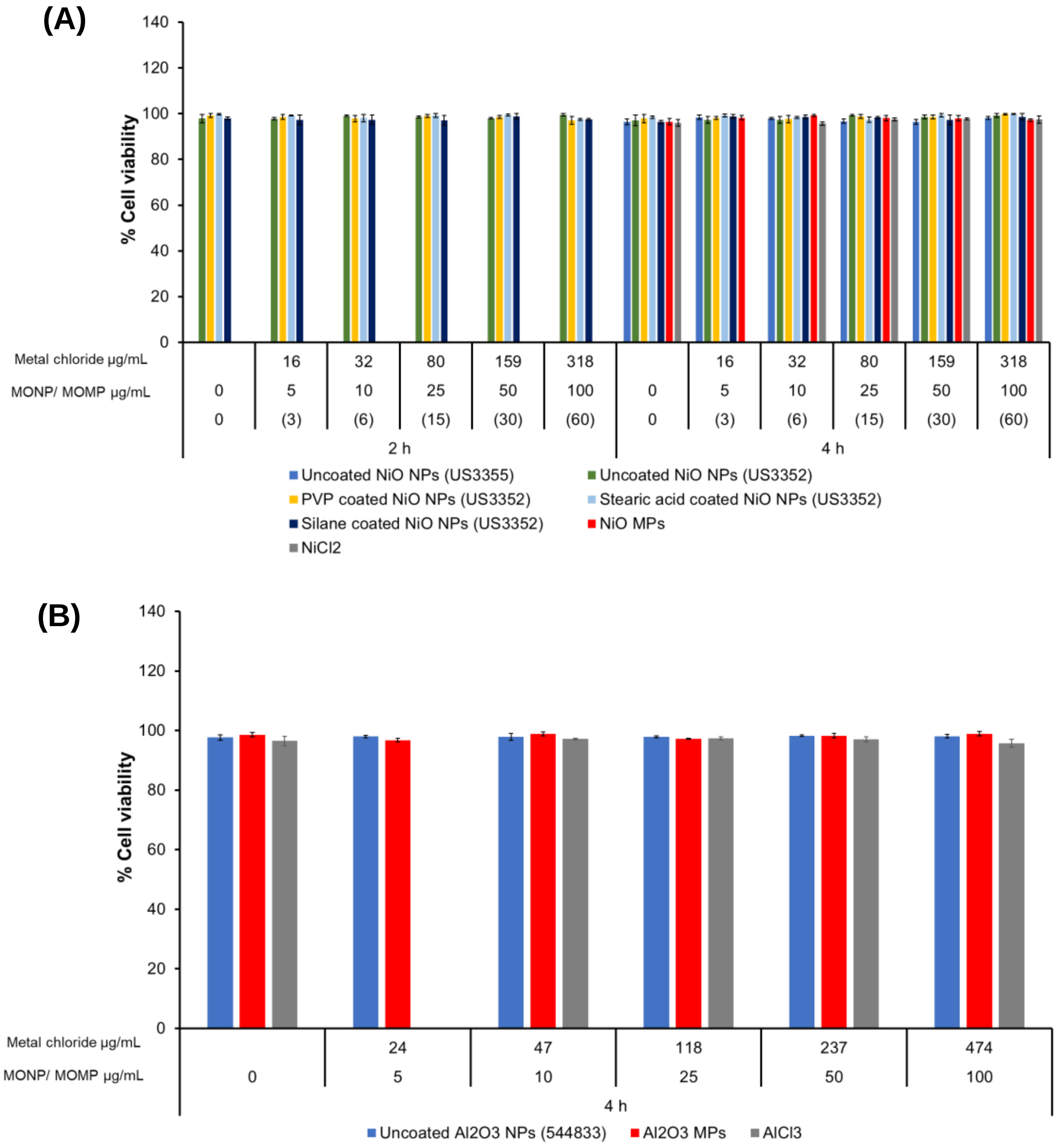

Figure S11. Percentage of cell viability after exposure to (A) NiO variants and $NiCl_2$ for 2 and 4 h, (B) Al_2O_3 variants and $AlCl_3$ for 4 h. Trypan Blue exclusion method. (A) Data in parenthesis indicates the concentration of stearic acid coated NiO NPs. Data is presented as mean and standard error (n=3-4 independent experiments). Statistically significant differences between the exposed samples and the matched negative control were determined through Kruskal-Wallis test.

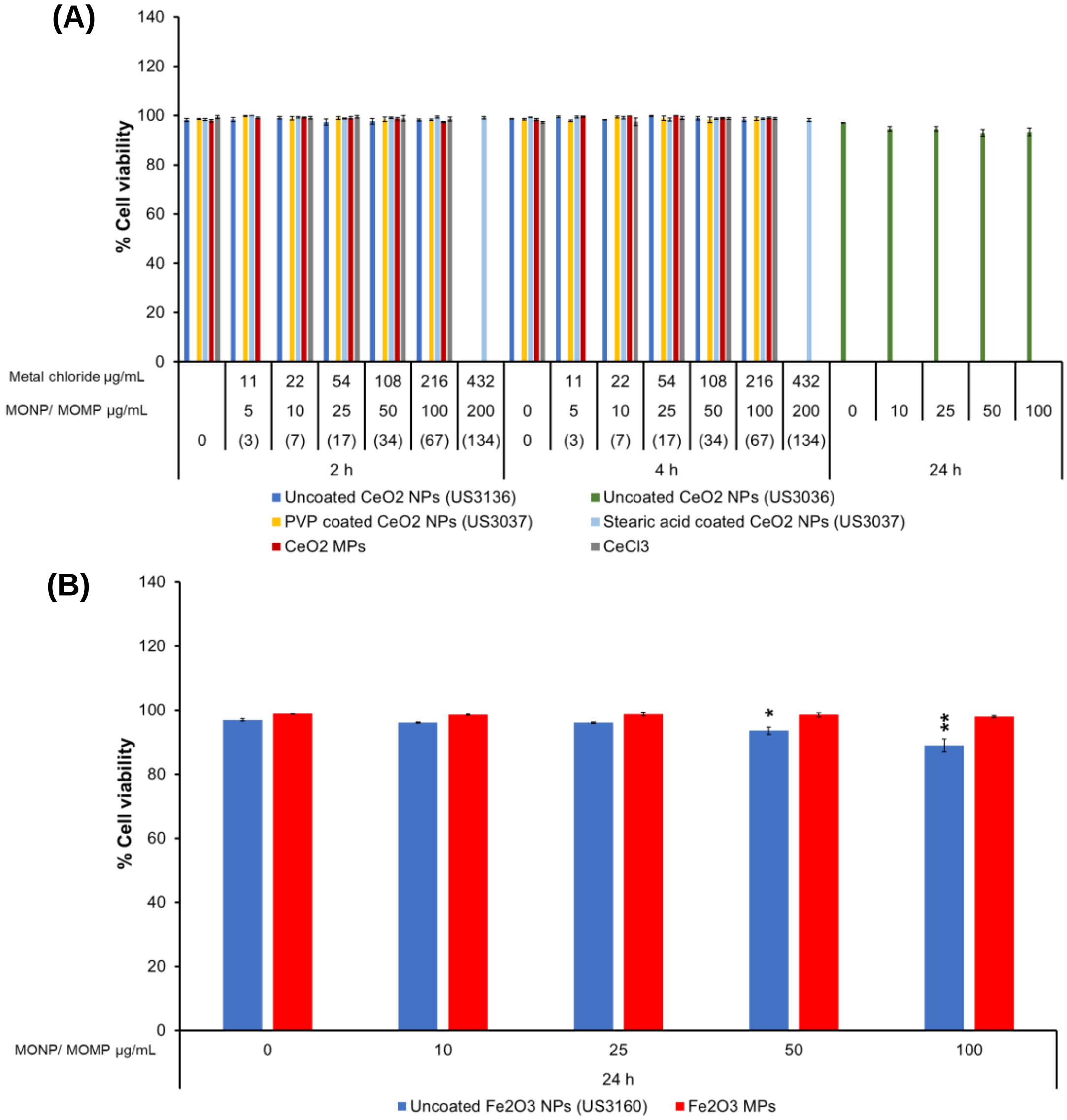

Figure S12. Percentage of cell viability after exposure to (A) CeO_2 variants and $CeCl_3$, (B) Fe_2O_3 variants. Uncoated CeO_2 NPs (US3036) and Fe_2O_3 variants were only evaluated at 24 h. Uncoated CeO_2 (US3136), coated CeO_2 NPs and $CeCl_3$ were evaluated after 2 and 4 h of exposure. Trypan Blue exclusion method. (A) Data in parenthesis indicates the concentration of stearic acid coated CeO_2 NPs. Data is presented as mean and standard error (n=3-4 independent experiments). Statistically significant differences between the exposed samples and the matched negative control were determined through Kruskal-Wallis test with a Dunnett's post-hoc. * $p < 0.05$, ** $p < 0.01$.

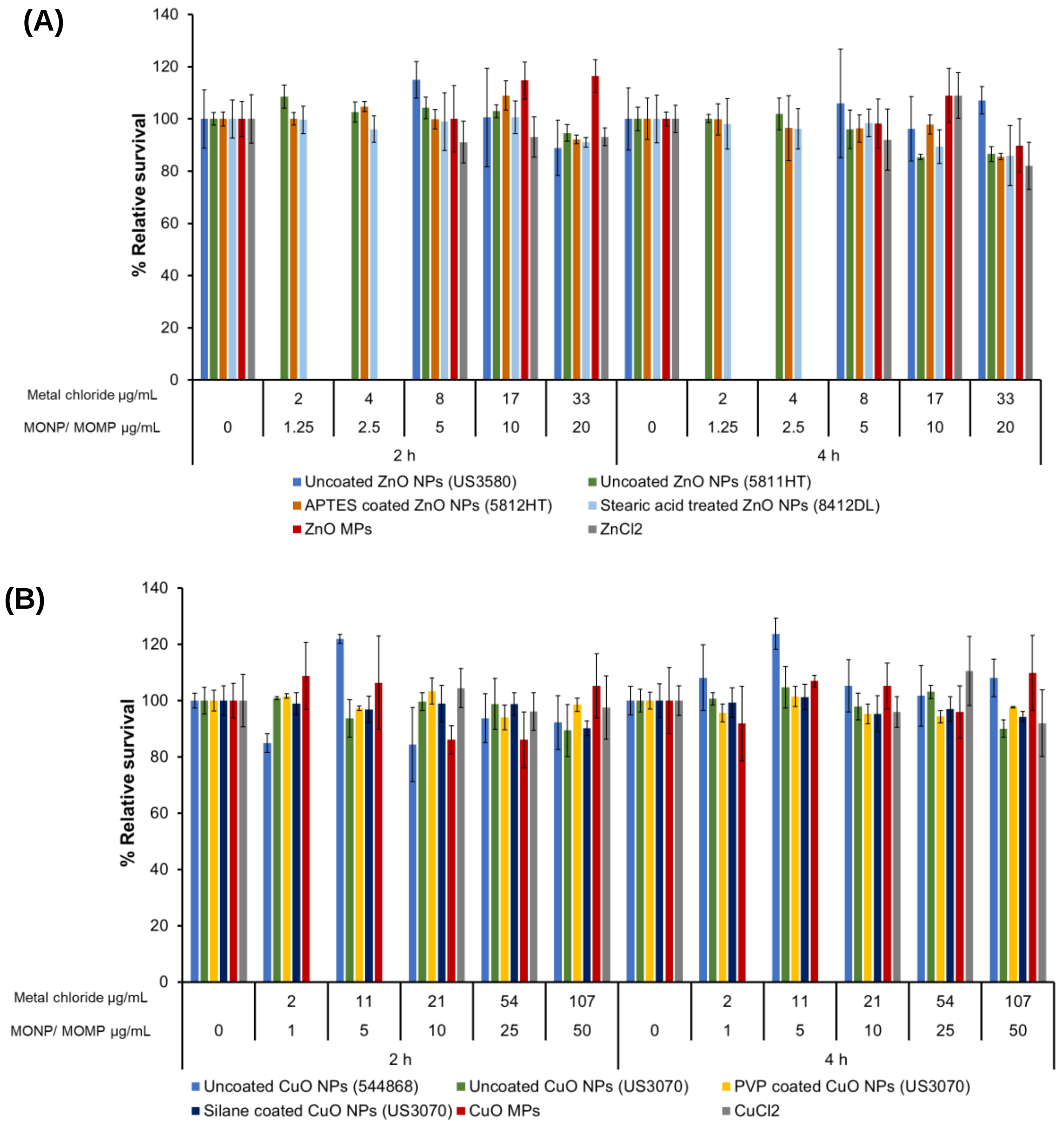

Figure S13. Percentage of relative survival after exposure to (A) ZnO variants and $ZnCl_2$, (B) CuO variants and $CuCl_2$. Trypan Blue exclusion method was conducted after 2 and 4 h of exposure. Data is presented as mean and standard error (n=3-4 independent experiments). Statistically significant differences between the exposed samples and the matched negative control were determined through Kruskal-Wallis test.

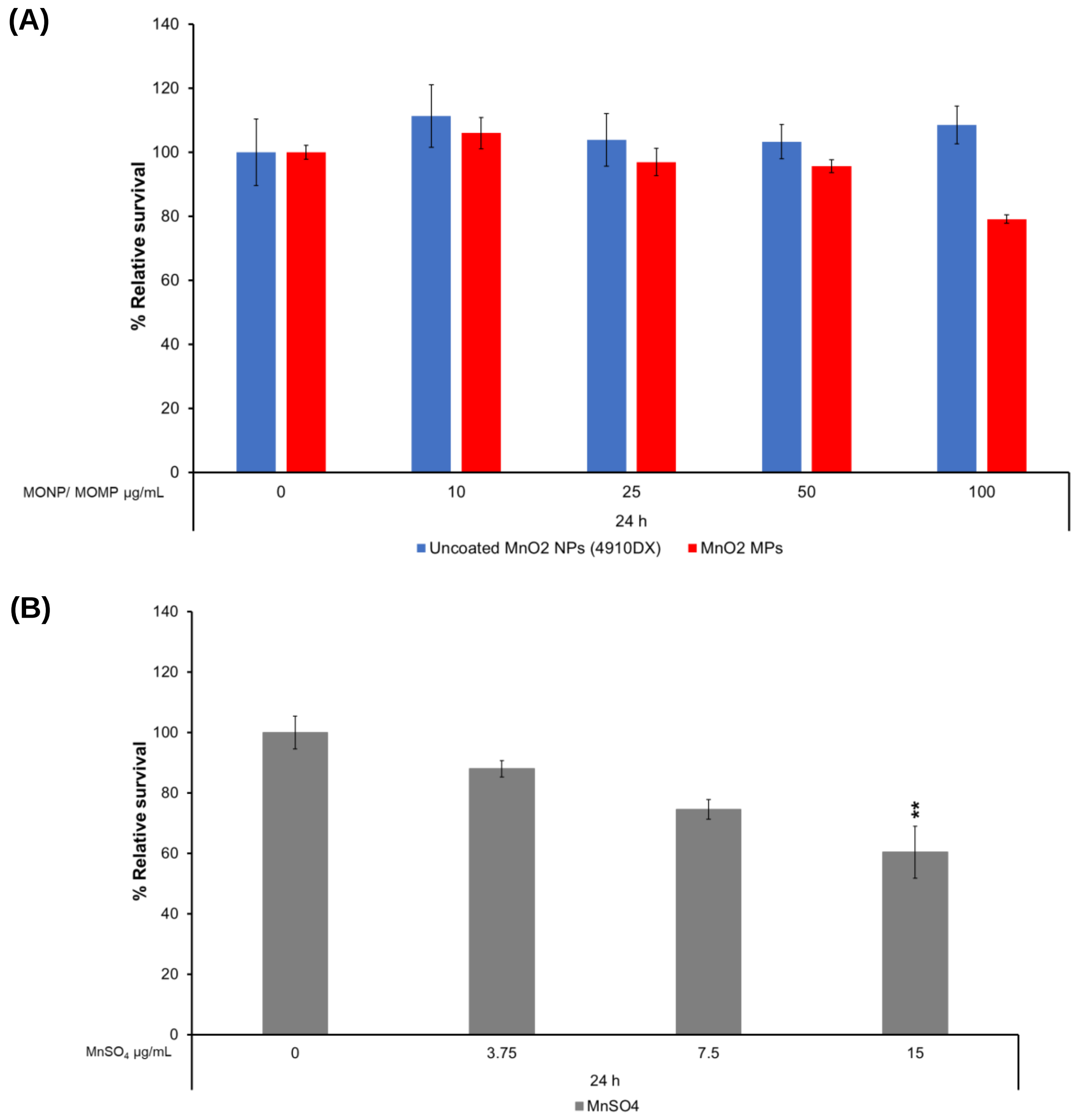

Figure S14. Percentage of relative survival after exposure to (A) MnO_2 variants and $MnSO_4$ for 24 h. Trypan Blue exclusion method. Data is presented as mean and standard error (n=3-4 independent experiments). Statistically significant differences between the exposed samples and the matched negative control were determined through Kruskal-Wallis test with a Dunnett's post-hoc. ** $p < 0.01$.

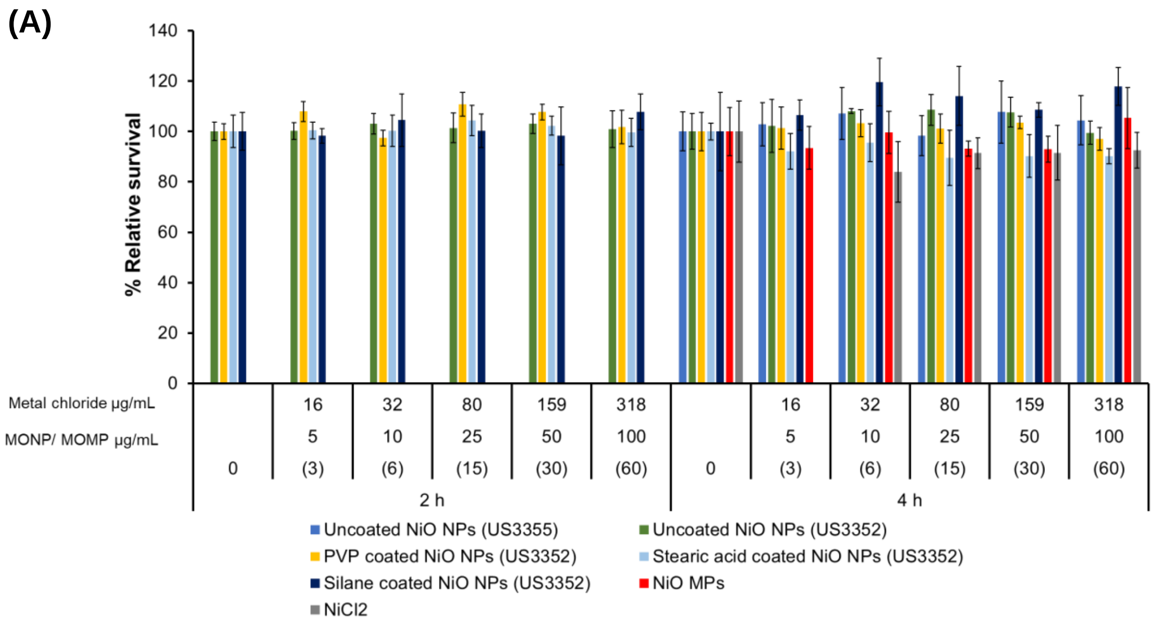

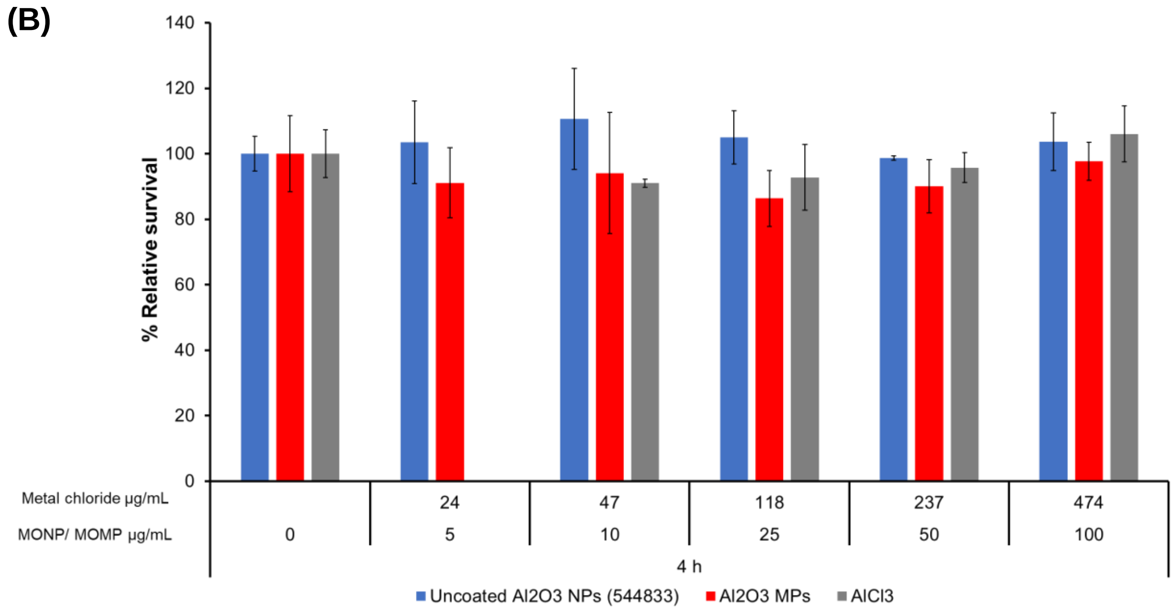

Figure S15. Percentage of relative survival after exposure to (A) NiO variants and $NiCl_2$ for 2 and 4 h, (B) Al_2O_3 variants and $AlCl_3$ for 4 h. Trypan Blue exclusion method. (A) Data in parenthesis indicates the concentration of stearic acid coated NiO NPs. Data is presented as mean and standard error (n=3-4 independent experiments). Statistically significant differences between the exposed samples and the matched negative control were determined through Kruskal-Wallis test.

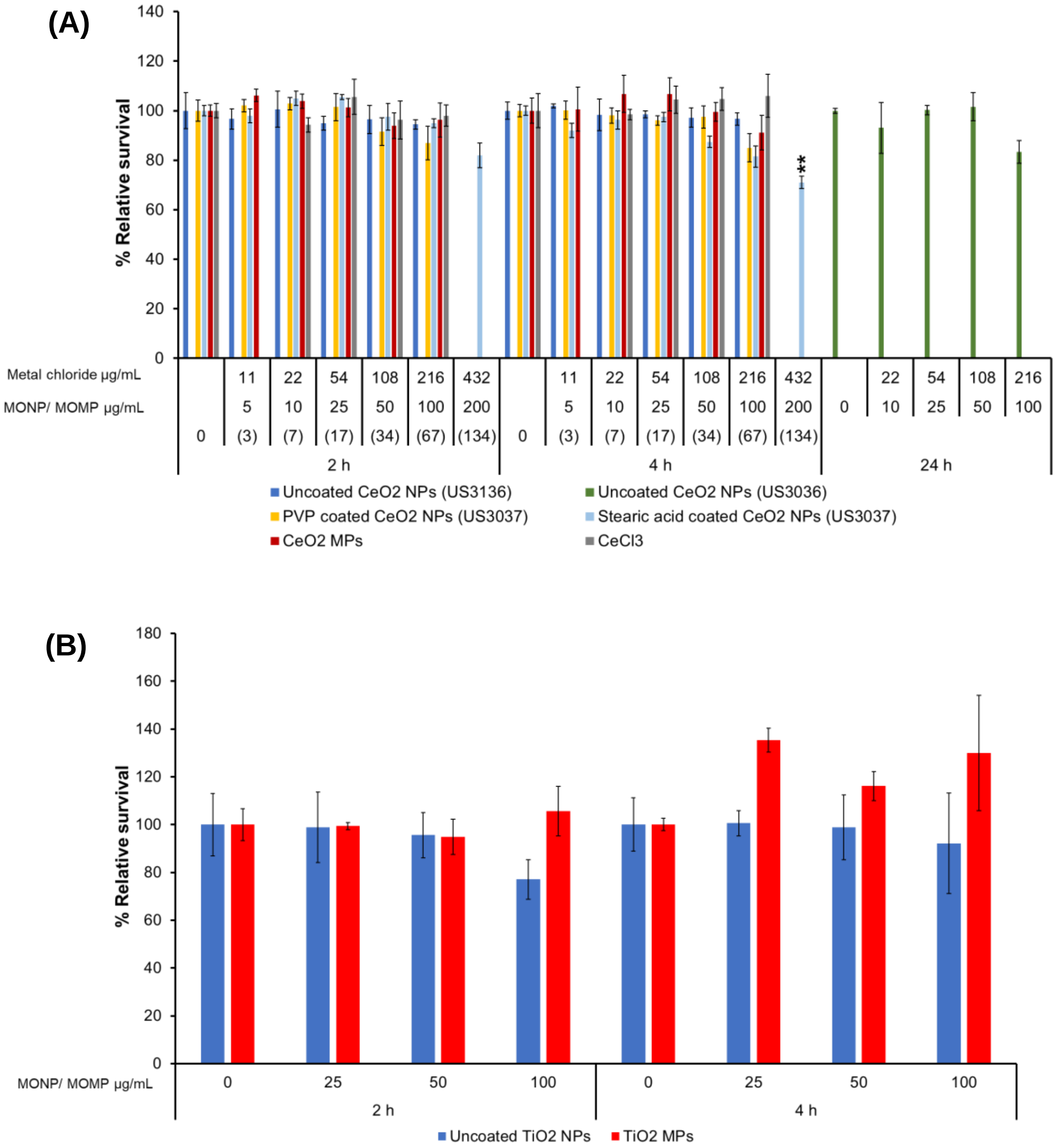

Figure S16. Percentage of relative survival after exposure to (A) CeO_2 variants and $CeCl_3$ and (B) TiO_2 variants for 2 and 4 h. Uncoated CeO_2 NPs (US3036) were only evaluated after 24 h of exposure. Trypan Blue exclusion method. (A) Data in parenthesis indicates the concentration of stearic acid coated CeO_2 NPs. Data is presented as mean and standard error (n=3-4 independent experiments). Statistically significant differences between the exposed samples and the matched negative control were determined through Kruskal-Wallis test with a Dunnett's post-hoc. ** $p < 0.01$.

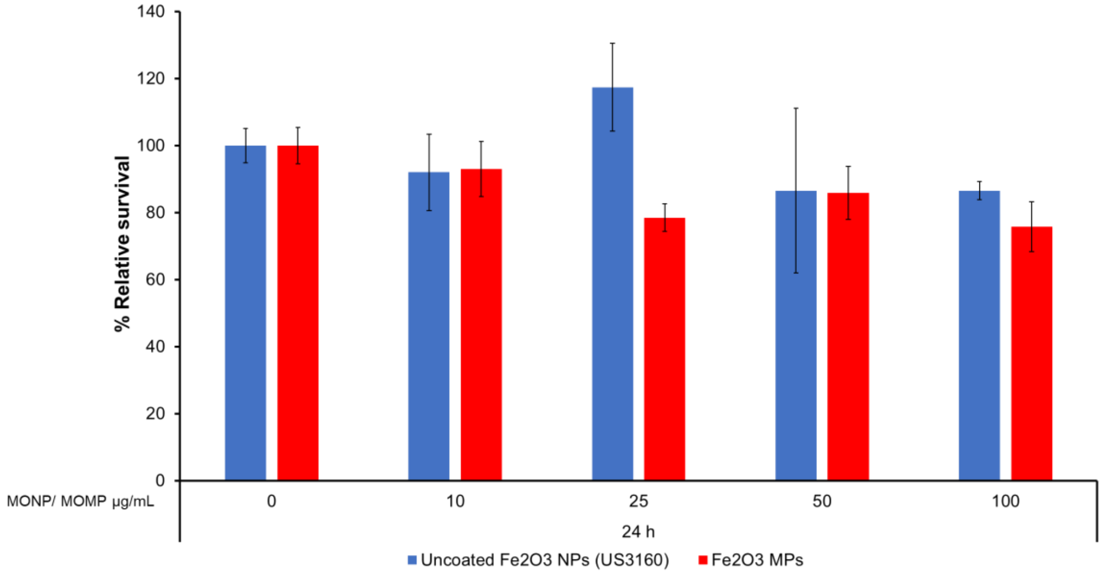

Figure S17. Percentage of relative survival after exposure to (A) Fe_2O_3 variants for 24 h. Trypan Blue exclusion method. Data is presented as mean and standard error (n=3-4 independent experiments). Statistically significant differences between the exposed samples and the matched negative control were determined through Kruskal-Wallis test.

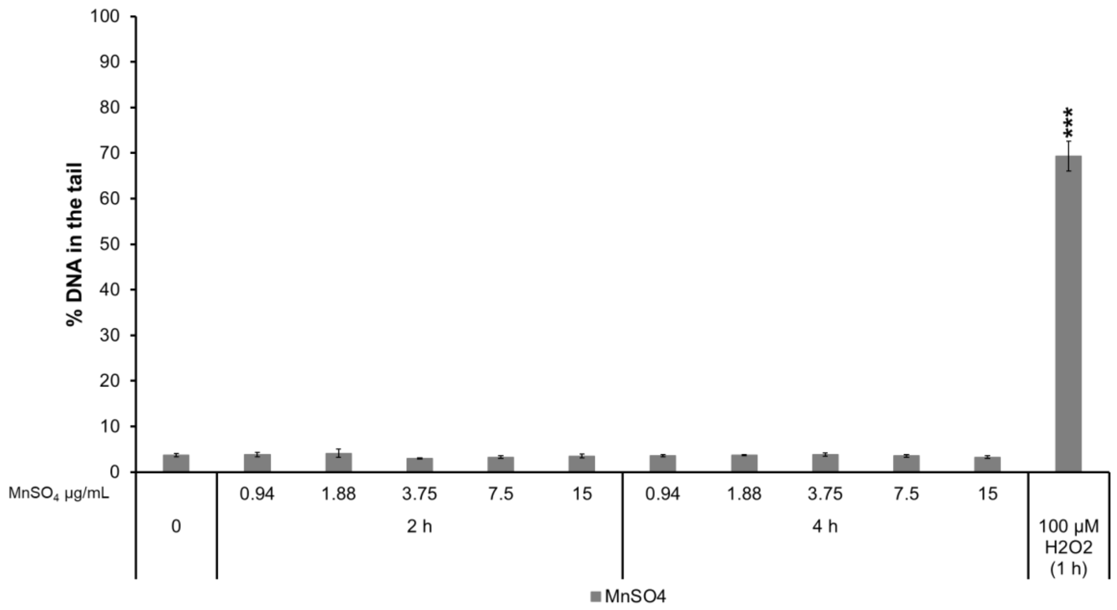

Figure S18. Percentage of DNA in the tail in FE1 cells after exposure to $MnSO_4$ at 2 and 4 h. Data is presented as mean and standard error (n=3-4). Statistically significant differences between the exposed samples and the matched negative control were determined through one-way ANOVA with a Dunnett's post-hoc. *** $p < 0.001$.

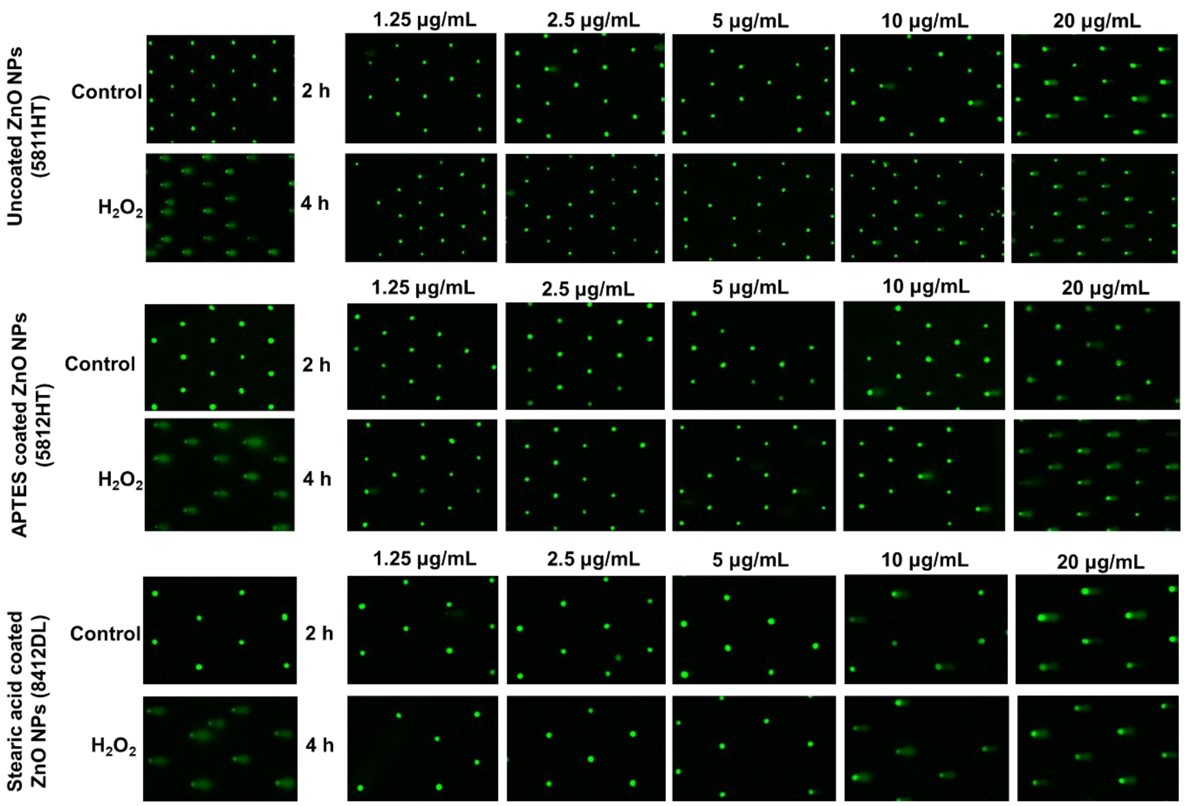

Figure S19. Representative images of comets after exposure to uncoated ZnO NPs (5811HT), APTES coated ZnO NPs (5812HT), and stearic acid coated ZnO NPs (8412DL). SYBR® Gold staining. Leica DMi8 automated confocal fluorescence microscope 5×.

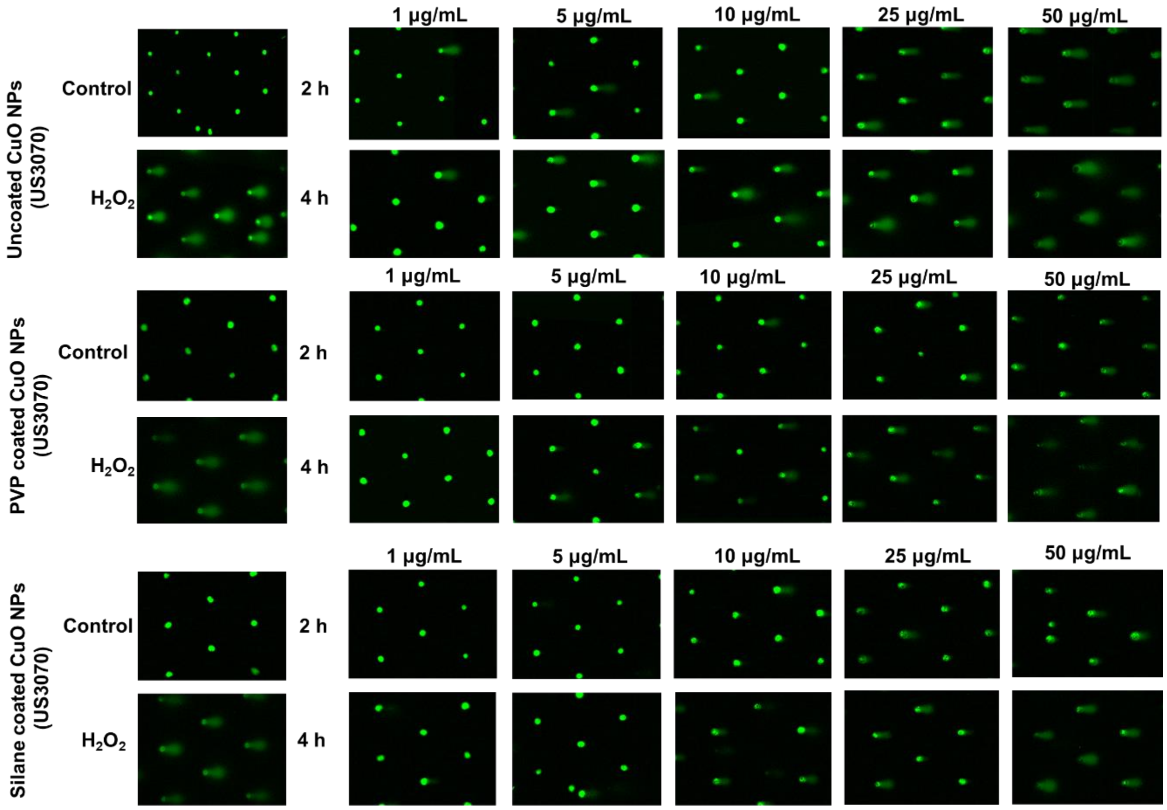

Figure S20. Representative images of comets after exposure to uncoated CuO NPs (US3070), PVP coated CuO NPs (US3070), and silane coated CuO NPs (US3070). SYBR® Gold staining. Leica DMi8 automated confocal fluorescence microscope 5×.

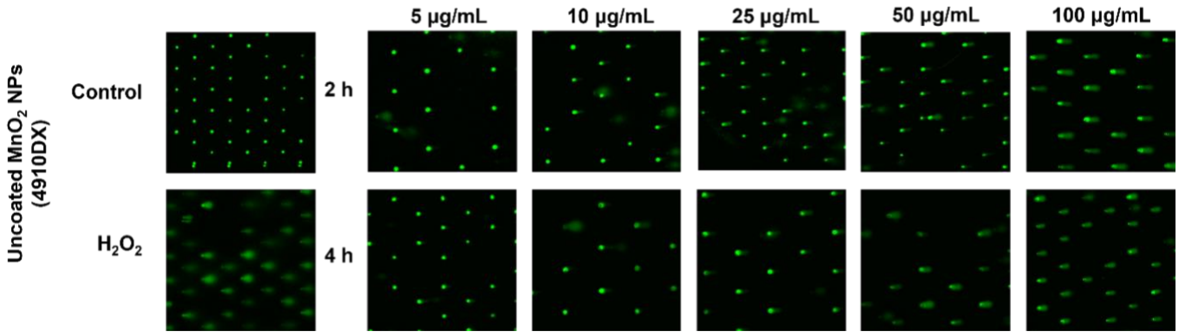

Figure S21. Representative images of comets after exposure to uncoated MnO_2 NPs (4910DX). SYBR® Gold staining. Leica DMi8 automated confocal fluorescence microscope 5×.

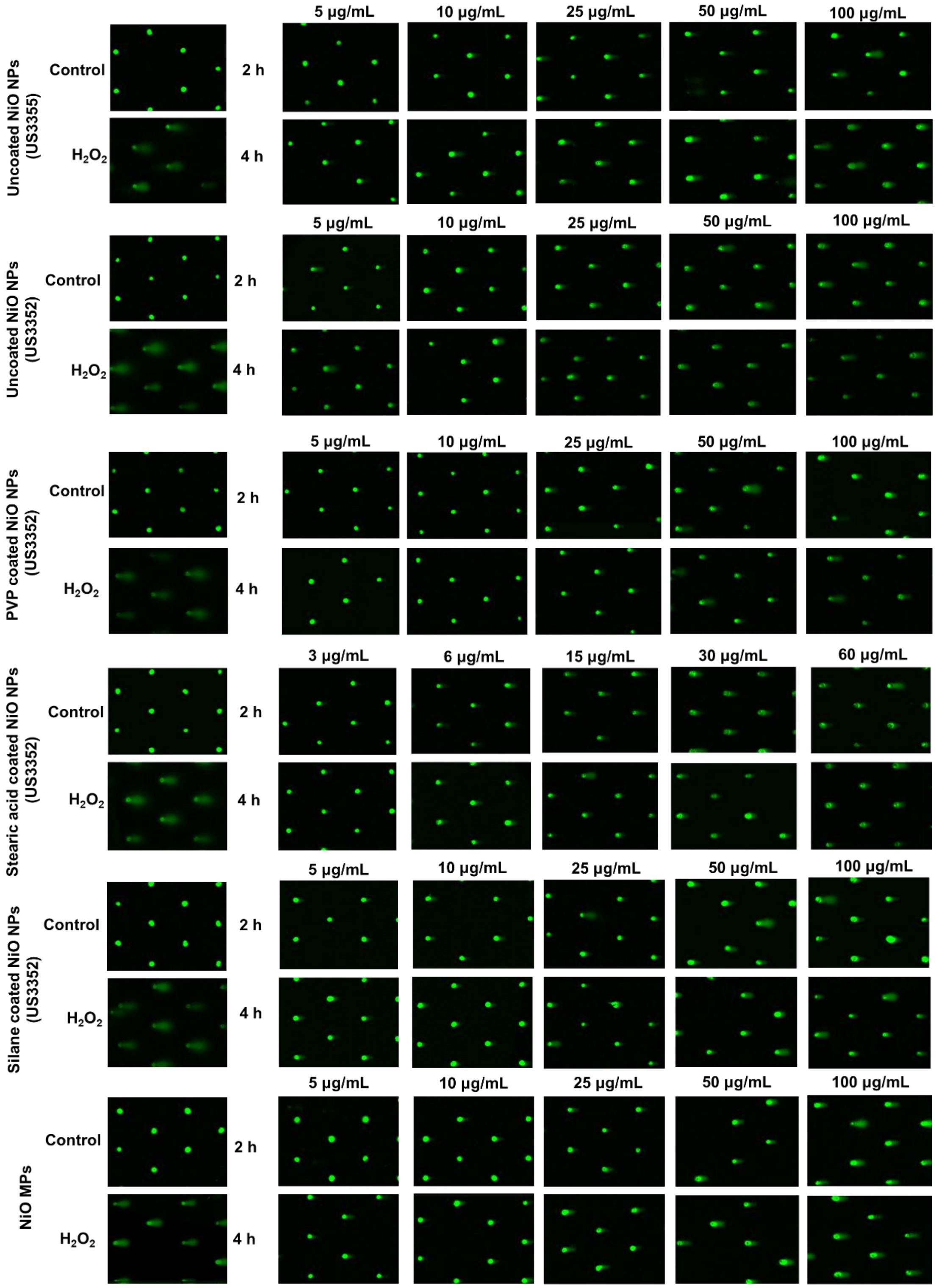

Figure S22. Representative images of comets after exposure to NiO variants. SYBR® Gold staining. Leica DMi8 automated confocal fluorescence microscope 5×.
